# GLI1 inhibitor GANT61 exhibits antitumor efficacy in T-cell lymphoma cells through down-regulation of p-STAT3 and SOCS3

**DOI:** 10.18632/oncotarget.9792

**Published:** 2016-06-02

**Authors:** Lingyun Geng, Kang Lu, Peipei Li, Xinyu Li, Xiangxiang Zhou, Ying Li, Xin Wang

**Affiliations:** ^1^ Department of Hematology, Shandong Provincial Hospital Affiliated to Shandong University, Jinan 250021, P.R. China

**Keywords:** T-cell lymphoma, GLI1, STAT3, signaling pathway, targeted therapy

## Abstract

T-cell lymphomas are lymphoid malignancies with aggressive clinical course and poor prognosis. Increasing evidences suggest that deregulation of signal transducer and activator of transcription-3 (STAT3) and suppressor of cytokine signaling 3 (SOCS3) is associated with the pathogenesis of T-cell lymphomas. The hedgehog (Hh)/glioma-associated oncogene-1 (GLI1) pathway, aberrantly activated in a number of tumors, has also been extensively studied. We found that protein expressions of GL11, p-STAT3, STAT3, and SOCS3 were up-regulated in T-cell lymphoma tissues and cell lines. Moreover, the protein expressions of p-STAT3 and SOCS3 were positively correlated with GLI1 in T-cell lymphomas. GLI1 inhibitor GANT61 and lentivirus-mediated siGLI1 exhibited inhibitory effects in the three T-cell lines (Jurkat, Karpass299 and Myla3676 cells). The protein expressions of p-STAT3 and SOCS3 were decreased accompanied with the inhibition of GLI1. These findings indicated that GANT61 is a promising agent against T-cell lymphoma and the antitumor activity might be partly mediated by down-regulating p-STAT3 and SOCS3.

## INTRODUCTION

T-cell lymphomas are a heterogeneous group of lymphoid malignancies committed to the T-cell lineage. Compared with B-cell lymphomas, T- cell are more aggressive with poorer prognosis [1]. The etiology remains to be elucidated, and no uniformed therapeutic strategies have been achieved. Novel effective agents are urgently needed. Accumulating lines of evidence suggests that the improper activation of signal transduction pathways (e.g. PI3K/AKT, Hedgehog, STAT3) are critical events in pathologies of T-cell lymphomas [2, 3]. The intermediates in these pathways contributing to the tumorgenesis are attractive therapeutic targets. This study mainly focuses on the Hh/GLI1 and STAT3/SOCS3 pathways in T-cell lymphomas.

The hedgehog (Hh) signaling pathway is a conserved developmental pathway [4]. Enhanced Hh signaling has been described in more than 30% of human cancers, including glioma, pancreatic cancer, lymohoma, leukemia [5–8]. Briefly, the pathway is mainly composed of HH ligands, the ligand-binding subunit ptched (PTCH) and signal transduction subunit smoothened (SMO), transcription factors (GL11-3) and target genes [9]. Activated GLI transcription factors ultimately translocate into the nucleus to activated target genes such as GLI1, PTCH, CyclinD1, Bcl-2, C-Myc, VEGF [10, 11]. Since GLI1 is both a transcription factor and a target gene of Hh signaling, the expression of GLI1 is often used as the readout of Hh pathway activation. GANT61, a promising GLI1 inhibitor, appears to be highly effective against human malignant cells and in xenograft mouse models by targeting almost all of the classical hallmarks of cancer [12, 13].

STAT3 is a central cytoplasmic transcription factor in response to cytokines and growth factors, such as IL-6, IL-2, epidermal growth factor (EGF), fibroblast growth factor (FGF) [14]. In the cascade reaction, STAT3 proteins are phosphorylated by Tyr kinases of growth factor receptors, or by cytoplasmic non-receptor Tyr kinases. Subsequently, phosphorylated STAT3 proteins dimerize and translocate into the nucleus to regulate the genes involved in cell proliferation and survival. Normally, STAT3 activation is rapid and transient. However, inappropriate activation of STAT3 has been implicated in a wide range of hematological malignancies, such as leukemia and diffuse large B cell lymphoma (DLBCL) [15, 16]. The p-STAT3 is used as a measure of activation of STAT3. SOCS3 are STAT3 induced negative regulators of cytokine-induced signaling pathways in a negative-feedback manner. Constitutive expression of SOCS3 has been documented in AML, follicular lymphoma [17, 18].

Aberrant activation of Hh/GLI1 and STAT3 pathways usually occur in tumors, however their crosstalk is quite unknown in T cell lymphomas. STAT3 is a well-known mediator activated by protective growth factors and cytokines to regulate cell growth by inducing pro-survival genes. It is unclear whether the pro-survival or protective potential of Hh signaling is mediated by activating STAT3 pathway in T cell lymphomas. In this study we investigated the antitumor effect and elucidate the possible cytostatic mechanism of GANT61 in T-cell lymphoma cells.

## RESULTS

### Elevated levels of GLI1, p-STAT3 (Tyr705), STAT3 and SOCS3 proteins both in T-cell lymphoma tissues and T-cell lines

**Figure 1:**
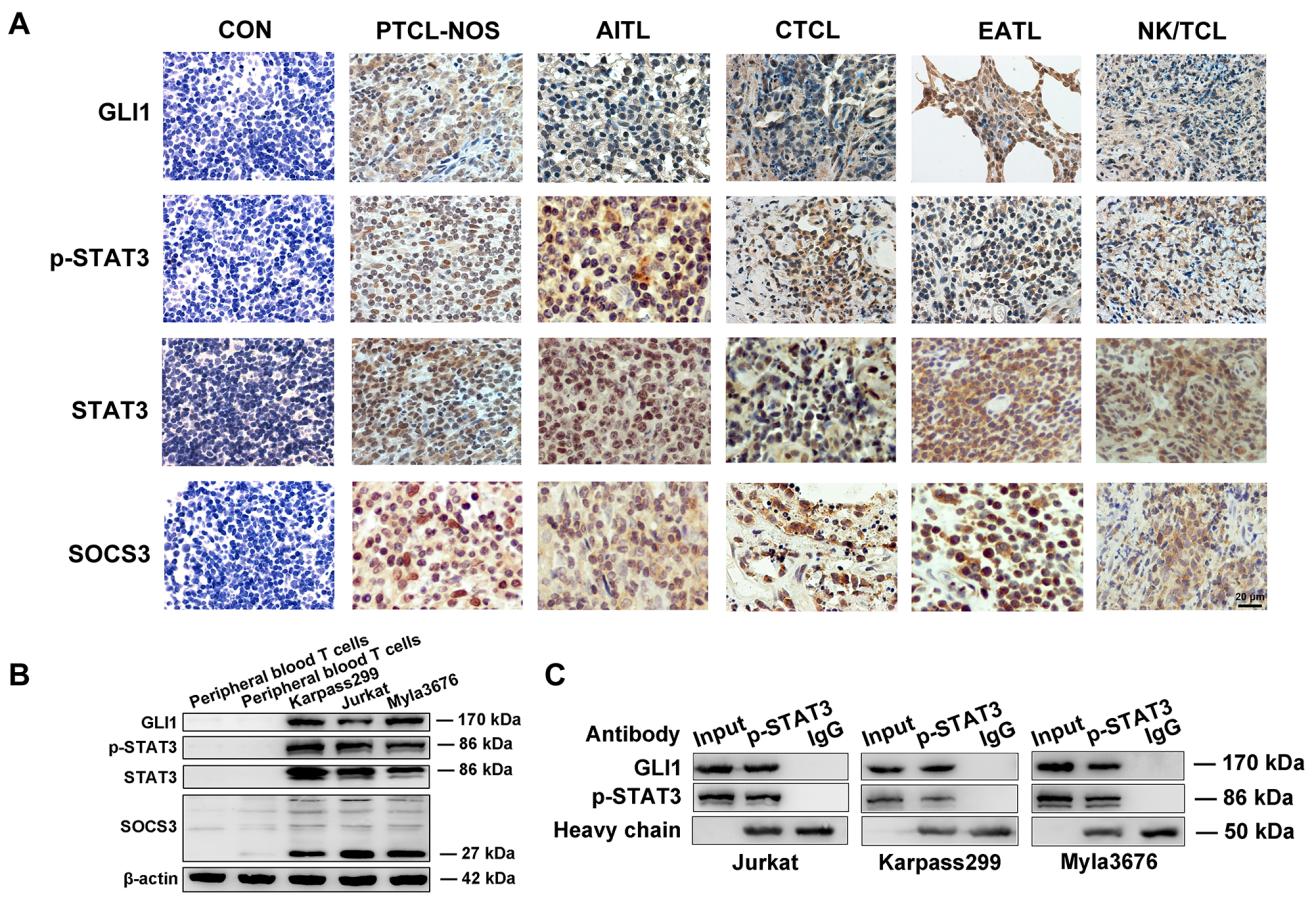
Elevated levels of GLI1, p-STAT3, STAT3 and SOCS3 proteins in T-cell lymphoma samples and T-cell lines **A**. Represent images of immunohistochemistry showing elevated expression levels of GLI1, p-STAT3, STAT3, and SOCS3 in T-cell lymphomas. Reactive hyperplasia of lymph node (RHL) served as control samples. The Scale bar= 20 μm. **B**. Western blot analysis revealed higher expression of GLI1, p-STAT3, STAT3, and SOCS3 in Jurkat, karpass299, and myla3676 cells, compared with peripheral T cells from healthy volunteers. **C**. The interaction of GLI1 and p-STAT3 in the three cells were observed in the western blot analysis of Co-Immunoprecipitation (Co-IP). (PTCL-NOS, peripheral T-cell lymphoma, non-specific; AITL, angioimmunoblasic T-cell lymphoma; CTCL, cutaneous T-cell lymphoma; EATL, enteropathy associated T-cell lymphoma)

We examined the expression of GLI1, p-STAT3, STAT3 and SOCS3 for tissues from 35 cases of T-cell lymphomas by immunohistochemistry, in comparation with the control of 15 cases of reactive hyperplasia of lymph node (RHL). Positive staining of the three proteins were highly elevated in most T-cell lymphomas samples but too weak to be detected in the RHL tissues (Figure 1A). The expressions of GLI1, p-STAT3, STAT3, and SOCS3 were detected respectively in 28/35(80.0%), 26/35(74.2%), 32/35(91.4%), and 24/35(68.6%) of T-cell lymphoma cases, when tumors displaying staining in 30% or more of the cells were categorized as positive cases. More detailed data were summarized in Table 1. Additionally, GLI1, p-STAT3, STAT3, and SOCS3 were detected both in the tumor and the adjacent stromal tissues, indicating that the involvement of these proteins in the tumor microenvironment.

**Table 1:** Immunohistochemical expressions of GLI1, p-STAT3, STAT3, and SOCS3 in T-cell lymphoma tissues

	PTCL-NOS (*n*=12)	AITL (*n*=6)	CTCL (*n*=6)	EATL (*n*=4)	NK/TCL (*n*=7)
GLI1					
Positive	10 (83.3%)	5 (83.3%)	4 (66.7%)	4(100.0%)	5 (71.4%)
Negative	2 (16.7%)	1 (16.7%)	2 (33.3%)	0 (0.0%)	2 (28.6%)
p-STAT3					
Positive	8 (66.1%)	4 (66.7%)	5 (83.3%)	4(100.0%)	5 (71.4%)
Negative	4 (33.3%)	2 (33.3%)	1 (16.7%)	0 (0.0%)	2 (28.6%)
STAT3					
Positive	11 (91.7%)	5 (83.3%)	6 (100.0%)	4(100.0%)	6 (85.7%)
Negative	1 (8.3%)	1 (16.7%)	0 (0.0%)	0 (0.0%)	1 (14.3%)
SOCS3					
Positive	9 (75.0%)	4 (66.7%)	5 (83.3%)	3(75.0%)	3 (50.0%)
Negative	3 (25.0%)	2 (33.3%)	1 (16.7%)	1(25.0%)	3 (50.0%)

Positive, cells displaying staining accounted for more than 30%;

Negative, cells displaying staining accounted for 30% or less.

Western blot analysis also showed higher expressions of GLI1, p-STAT3, STAT3, and SOCS3 in three T-cell lines (Jurkat, Karpass299 and Myla3676 cells) compared to peripheral blood T lymphocytes freshly isolated from healthy donors (Figure 1B). The results confirmed our immunofluorescent results (Supplementary Figure S1).

### Correlation between Hh/GLI1 and STAT3/SOCS3 pathways in T-cell lymphomas

In order to explore the potential connections between Hh/GLI1 and STAT3/SOCS3 pathways in T-cell lymphomas, those immunohistochemical results were incorporated into the Fisher's exact probability test. Analysis results showed that both p-STAT3 and SOCS3 protein expression were positively correlated with GLI1 in T-cell lymphomas with the P value as 0.06 and 0.021 respectively (Table 2). We speculated that activation of GLI1 could activate p-STAT3 and SOCS3 expression directly or indirectly, and thereby activated Hh signaling resulting in T-cell lymphoma cell ontogeny. What's more, the interaction of GLI1 and p-STAT3 in Jurkat, Myla3676 and Karpass299 cells have been observed in the Western blot analysis of Co-Immunoprecipitation (Co-IP) (Figure 1C).

**Table 2:** Relationship between GLI1 and p-STAT3 or SOCS3 protein expressions in tissues from 35 cases of T-cell lymphomas

Protein expression	GLI1	
	Positive (*n*=28)	Negative (*n*=7)
p-STAT3*		
Positive	24 (85.7%)	2 (28.5%)
Negative	4 (14.3%)	5 (71.5%)
SOCS3*		
Positive	22 (78.5%)	2 (28.5%)
Negative	6 (21.4%)	5 (71.5%)

*P < 0.05, Fisher's exact probability test.

### GANT61 inhibited proliferation and induced apoptosis of T-cell Lines

**Figure 2:**
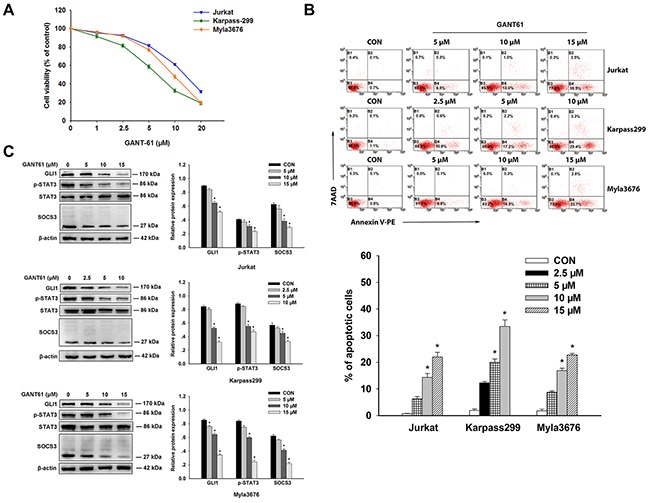
Antitumor effects of GANT61 on T-cell lines **A**. Cultivation with GANT61 for 48 hours at different concentrations (2.5, 5, 10, 20μM) inhibited the proliferation of Jurkat, karpass299, and Myla3676 cells in a dose-dependent manner. Viability of these cells were evaluated by CCK-8 assay. **B**. After exposure to GANT61 of different concentrations or 0.1% solvent (DMSO) for 24 hours, apoptosis of the three cells were quantified by annexin V/7-AAD flow cytometry. The number of apoptosis cells increased in a dose-dependent manner with the GANT61 treatment. **C**. Western blot revealed that the expression levels of p-STAT3, and SOCS3 decreased accompanied with the inhibition of GLI1 by GANT61 in the three cells. Statistical data represent the means ± SEM of three independent experiments. (*P < 0.05, compared with solvent group).

Cytotoxicity of GANT61 on T-cell lines (Jurkat, Karpass299 and Myla3676 cells) was assessed by the cell counting kit-8 (CCK-8) assay after a 48-hour GANT61 treatment at different concentrations. Viability of the three cell lines decreased in a dose-dependent manner after 48h incubation with different concentrations of GANT61 (Figure 2A). The half-maximal inhibitory concentration (IC50) values of GANT61 at 48h were calculated as following: Jurkat cells, 13.761±0.81μM; Karpass299 cells, 6.81±0.91μM; and Myla3676 cells, 10.23±0.94 μM. Effect of GANT61 on apoptosis of these cells was evaluated by AnexinV-PE/7AAD assay, and protein expression of GLI1, p-STAT3, STAT3 and SOCS3 was detected by Western-blot after a 24-hour GANT61 treatment at different concentrations. The percentage of apoptotic cells increased significantly at 24 h following medium to high-concentration GANT61 treatment compared with the solvent treatment groups (control groups) (*P*<0.05) (Figure 2B). Through the analysis of western blots results in the three cells after a 24-hour treatment of GANT61 (Figure 2C), we found that both 10μM and 15μM of GANT61 markedly inhibited GLI1 in Jurkat cells (*P*<0.05); both 5μM and 10μM of GANT61 markedly inhibited GLI1 in Karpass299 cells (*P*<0.05); 5μM, 10μM, and 15μM of GANT61 all inhibited GLI1 significantly (*P*<0.05). These data demonstrated that the expression of GLI1 is dose dependent on the inhibitor. Furthermore, the degrees of reduction in p-STAT3 and SOCS3 were in accordance with the inhibition GLI1 in the three cells (*P*<0.05) (Figure 2C), indicating that p-STAT3 and SOCS3 protein were positively associated with GLI1.

### Effects of silencing of GLI1 expression on T-cell lines

**Figure 3:**
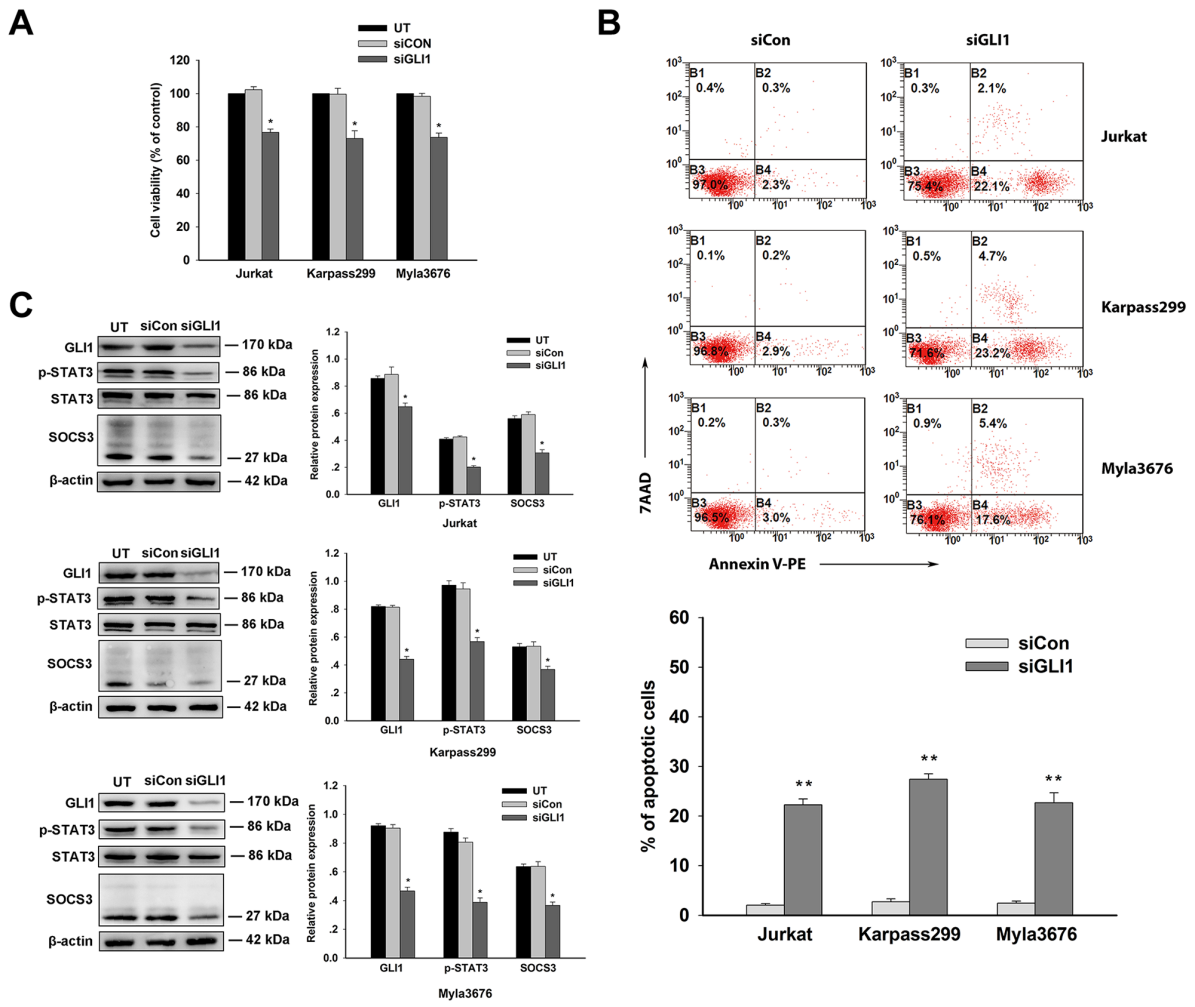
Effects of silencing of GLI1 expression on T-cell lines **A**. Survival of Jurkat, karpass299, and Myla3676 cells transfected with siGLI1 were measured by CCK-8 assay. Negative control siRNA transfected cells served as control group. There was no difference on viability between negative control siRNA transfected or untransfected cells statistically. **B**. From the annexin V/7-AAD flow cytometry, increased apoptosis were observed in GLI1-silenced cells compared with negative control siRNA transfected cells (**P<0.01). **C**. Western blots were performed using the antibodies indicated to analyze expression levels of GLI1, p-STAT3, and SOCS3 of these cells after transfected with GLI1 siRNA or negative control siRNA. Statistical data represent the means ± SEM of three independent experiments. (*P < 0.05, **P < 0.01, compared with siCon group).

To confirm the hypothesis that targeting GLI1 is of critical therapeutic value in T-cell lymphomas, lentivirus-mediated RNA interference was carried out to specifically knockdown GLI1 expression in the three cell lines (Jurkat, Karpass299 and Myla3676 cells). CCK8 assay showed all the three stable siGLI1 transfected cells exhibited decreased viability compared with negative control siRNA transfected cells (*P*<0.05) (Figure 3A). There was no difference between negative control siRNA transfected or untransfected cells statistically. Significantly increased apoptosis of the three siGli1 transfected cells were demonstrated by AnexinV/7AAD assay compared with control siRNA transfected cells (*P*<0.01) (Figure 3B). Effective silencing of GLI1 was evidenced by decreased GLI1 protein levels shown in western blot analysis. Notably, silencing of GLI1 expression also brought about the reduction of protein expression of p-STAT3 and SOCS3 compared with negative control siRNA transfected cells (*P*<0.05) (Figure 3C). These data showed the specific role of GLI1 in the pathogenesis. Pharmacological inhibition or genetic interference targeting GLI1 provided potential therapeutic strategies to improve the poor prognosis of T-cell lymphomas.

## DISCUSSION

Our study results showed that protein expressions of GL11, p-STAT3, STAT3, and SOCS3 were up-regulated both in T-cell lymphoma tissues and T-cell lines. Inhibition of GL11 by GANT61 and RNA interference could attenuate proliferation and induce apoptosis of Jurkat, Karpass299 and Myla3676 cells. The expression of GLI1 is dose dependent on the inhibitor. Moreover, the p-STAT3 and SOCS3 were decreased accompanied with the inhibition of GLI1. These data indicated a potential mechanism for the antitumor activity of GANT61 which might inhibit viability of T-cell lymphoma cells at least partially by down-regulating p-STAT3 and SOCS3.

GANT-61, an antagonists of GLI1, appears to be highly effective against human cancer cells *in vitro* and in xenograft mouse models [19–21]. In our study, the IC50 values of GANT61 at 48h were calculated as: Jurkat cells, 13.761±0.81μM; Karpass299 cells, 6.81±0.91μM; and Myla3676 cells, 10.23±0.94 μM. Agyeman et al. showed that the GANT61 causes the inhibition of GLI1-DNA binding and therefore GLI1-mediated transcription [22]. Until now, the exact working mechanism of GANT61 is largely unknown. Moreover, GANT61 serves as a valuable tool to investigate Hh pathway biology. Antitumor activity of GANT61 has been ascribed to its effect on cell viability, proliferation, apoptosis, DNA damage repair, autophagy, cancer stem cells and immune response [23–26]. Studying the mechanisms by which the GANT61 interacts with cancer cells is of great clinical interest for inhibiting growth, metastasis, and recurrence of cancers.

It was worth mentioning that Fisher's exact probability test of the immunochemical results showed both p-STAT3 and SOCS3 protein expression were positively correlated with GLI1 in T-cell lymphomas with each P value<0.05 (Table 2). The western blots analysis in the three cells after a 24-hour treatment of GANT61 also demonstrated the degrees of reduction in p-STAT3 and SOCS3 were in accordance with the inhibition GLI1 in the three cells (*P*<0.05), indicating that GLI1 may activate p-STAT3 and SOCS3 expressions directly or indirectly.

Although SOCS3 was described as a negative regulator of STAT3 in many malignancies, the exact role of SOCS3 in T-cell lymphoma was largely unknown. In our study, STAT3 and SOCS3 expression was simultaneously increased in T-cell lymphoma. We speculate about several possible mechanisms (Supplementary Figure S2). Firstly, there is negative-feedback between STAT3 and SOCS3, so aberrantly activated STAT3 stimulate the expression of SOCS3 in T-cell lymphoma. Secondly, SOCS3 may play an oncogenic role in T-cell lymphoma by inhibiting differentiation and apoptosis signals arising from specific cytokines.

As is known, inappropriate activation of STAT3 not only promotes the survival of the tumor cells, but also facilitates tumor migration, invasion and metastasis [27–30]. All of these rationales provided candidate mechanisms that could be investigated for the synergetic cooperation between Hh and STAT3 pathways. In the present study, the pro-survival transcription factor STAT3 was proved to be a new effector of Hh signaling-induced cellular protection in T cell lymphomas. These findings were consistent with a previous report that SMO agonist was sufficient to induce STAT3 phosphorylation at Tyr(705) in two HH-responsive cell lines ES14 and C3H10T1/2 [31]. Similarly, another study showed that loss of GLI1 led to decreased STAT3 activity in preneoplastic lesion of pancreatic model [6].

The physical interaction of GLI1 and p-STAT3 in the three T-cell lymphoma cells were demonstrated by Western blot analysis of Co-IP. Further investigations will be quite necessary to determine how the Hh and STAT3 pathways are linked. There are many hypothetical mechanisms to be testified in the future. For example, Hh signaling may activate STAT3 activity in transcription-dependent manner via interactions between STAT3 and GLI1 as reported in both the medulloblastoma and granule neuron precursor cells [32]. Alternatively, GLI1-dependent induction of cytokines or kinases (e.g. IL-6) may activate STAT3, which has been demonstrated in mouse pancreatic model [6].

Based on published data, Hh signaling may serve as the tumor driver, the tumor promoter, or the regulator for residual tumor cells during the development of various tumors. However, crosstalks among Hh and other oncogenic pathways were quite complex in the tumor microenvironment. Thus, recognizing the molecular alterations and designing rational combined-targeted therapy is significant in the era of targeted and personalized therapy. To the best of our knowledge, our study is the first to report the antitumor effect and working mechanism of GANT61 through down-regulation of STAT3 and SOCS3 in T cell lymphoma cells. This novel study would contribute to further investigation on the useful biomarkers and potential therapeutic target in the T-cell lymphomas. The crosstalk between Hh/GLI1 and STAT3/SOCS3 pathways deserves more exploration. GLI1 inhibitor and STAT3 inhibitor may be a promising combination to overcome or delay tolerance and decrease toxicity of single agent. The last but not the least, more preclinical and clinical trials of GANT61 are urgently desired to explore its pharmacokinetic, toxicity and so on.

## MATERIALS AND METHODS

### Cells lines and cell culture

Three T-cell lines (Jurkat, Karpass299 and Myla3676 cells) were used in the study. Jurkat (T-cell acute lymphoblastic leukemia cell line) was obtained from the Typical Culture Preservation Commission Cell Bank (Chinese Acadamy of Sciences, Shanghai. China). Karpass299 (ALK+ ALCL cell line) was purchased from Shanghai Bioleaf Biotech Company Limited. Myla3676 (Cutaneous T cell lymphocyte, lymphoblast, Sezary Syndrom) was retained by our laboratory. All cells were cultured at 37°C in RPMI-1640 (Gibco) supplemented with 10% heat-inactivated fetal bovine serum (FBS) (Gibco), 1% penicillin/streptomycin mixture, and 2mM L-glutamine in humidified atmosphere containing 5% CO2.

### Patients and samples

The paraffin-embedded tissue samples from 35 cases of T-cell lymphomas and 15 reactive hyperplasia of lymph node (RHL) were collected from Shandong Provincial Hospital affiliated to Shandong University prior to therapeutic intervention in this study. All patients were diagnosed according to the WHO criteria between January 2009 and July 2013. Of the 35 cases of T-cell lymphomas (23 males and 12 females; age range 25–82 years, median55 years), 12 were peripheral T-cell lymphoma non-specific (PTCL-NOS), 6 were angioimmunoblasic T-cell lymphoma (AITL), 6 were CTCL, 4 were enteropathy associated T-cell lymphoma (EATL), 7 were NK/TCL. Peripheral blood of healthy volunteers was drawn by heparin anticoagulation, and peripheral blood mononuclear cells (PBMCs) were obtained by Ficoll density gradient centrifugation (TBD Science, Tianjin, China). From this fraction, T cells were isolated by Nylon Wool Fiber Columns. This study was approved by the Medical Ethical Committee of Shandong Provincial Hospital affiliated to Shandong University. All human samples were obtained after informed consents had been given, according to the Declaration of Helsinki.

### Reagents

GLI1 inhibitor GANT61, purchased from Selleck Chemicals (Boston, MA, USA), was dissolved in DMSO to make the stock solution. Stock solution was finally diluted into cell culture medium to make the final working concentrations.

### Knockdown of human GLI1 in T lymphoma cells by RNA interference (RNAi)

RNAi target to human GLI1 gene used in this study was 5′- AAACGCTATACAGATCCTA -3′. The sequences of small interfering RNA (siRNA) targeting the sequence to human GLI1 gene and negative control siRNA were cloned into the pGCL-GFP and generated using stable lentivirus expression vectors by Shanghai GeneChem. T-cell lymphoma cells were plated in 96-well plates (10^4^cells/well) and were infected with GLI1-RNAi lentivirus or negative control lentivirus with the multiplicity of infection (MOI) 100 according to the manufacturer's instructions. The medium was changed with fresh medium after 8–12 hours. Infection efficiencies of these cells infected with lentivirus were determined via GFP fluorescence by flow cytometry. The stable transfected cells were then harvested for protein extraction or detection of cell viability and apoptosis.

### Immunohistochemistry (IHC)

Immunohistochemistry of patients' paraffin-embedded tissue sections was performed using primary rabbit antibodies for GLI1 (Abcam, Cambridge, MA, USA), p-STAT3 (Tyr705) (CST, Danvers, MA, USA), STAT3 (CST, Danvers, MA, USA), and SOCS3 (Abcam, Cambridge, MA, USA). Briefly, formalin-fixed, paraffin-embedded tissue sections of 4 μm thickness were deparaffinized and hydrated. High-pressure antigen retrieval was performed in 0.01M sodium citrate (PH 6.0). Endogenous peroxidase was blocked with 3% H_2_O_2_ in methanol for 15 minutes at room temperature, followed by incubation with normal serum to block non-specific staining. Primary rabbit antibodies were diluted as GLI1(1:200), p-STAT3 (Tyr705)(1:400), STAT3(1:400), SOCS3(1:100) and applied respectively to incubate the sections overnight at in a humidified chamber at 4°C; then the second antibody from SP reagent kit (Zhongshan Goldenbridge Biotechnology Company, Beijing, China) incubated the sections for 1 hour at room temperature. After washing, the tissue sections were treated with biotinylated anti-rabbit secondary antibody, followed by further incubation with streptavidin-horseradish peroxidase complex. After staining with diaminobenzidine Kit (DAB, Zhongshan Goldenbridge Biotechnology Company, Beijing, China), the sections were counterstained with hematoxylin and mounted. Immunohistochemical stainings were assessed in a series of randomly selected 5 high-power fields, which were believed to be representative of the average in tumors at ×400 magnification, by two independent observers who were blinded to all clinical data. The sections were scored according to the proportion of positively stained tumor cells. Tumors displaying staining in 30% or more of the cells were categorized as positive cases. In the meanwhile, tumors displaying staining less than 30% of the cells were categorized as negative cases.

### Western blot analysis

Total protein was extracted from peripheral blood T lymphocytes of healthy donors or T-cell lymphoma cells with designed treatment. Total protein was extracted using lysis buffer (Shenergy Biocolor, Shanghai, China) and 1% PhosSTOP (Roche, Mannheim, Germany). The protein concentration was determined by the BCA assay (Shenergy Biocolor, Shanghai, China). Cell lysate was then electrophoresed on 10% SDS-polyacrylamide gels and transferred onto polyvinylidene difluoride (PVDF) membranes. The membranes were blocked with 5% skim milk in Tris-saline buffer with 0.1% Tween-20, and then incubated with primary antibodies at 4°C overnight. After washing with TBST, secondary antibody conjugated with the horseradish peroxidase (Zhongshan Goldenbridge Biotechnology Company, China) was added to the membranes. Proteins were detected using the chemiluminescence detection kit (Millipore, USA). Primary antibodies against GLI1(1:1000), phospho-STAT3 (Tyr705) (1:2000) and STAT3(1:1000) were purchased from Cell Signaling Technology (Danvers, MA, USA). Primary antibody against SOCS3 (1:1000) was purchased from from Abcam. The expression level of β-ACTIN was used as the loading control for the western blot. Western blotting results were analyzed using the Las-4000 Image software and Multi Gauge Ver. 3.0 software (FujiFilm Life Science, Japan).

### Assessment of cell proliferation

The influences of GANT61 on viability of T-cell lymphoma cells were assessed by carrying out triplicate assays with the Cell Counting Kit-8 (CCK-8; Dojindo, Kumamoto, Japan). T-cell lymphoma cells (10000 cells/100 μl/well, respectively) were seeded into 96-well plates at 37°C with 5% CO_2_ and incubated with GANT61 at designed concentrations for 24 or 48 hours. Thereafter, the cells were incubated with 10uL of CCK-8 for 4 hours according to the manufacturer's recommendations. The absorbance at 450 nm was measured using a SpectraMax M2 Multi-Mode Microplate Reader (Molecular Devices, Sunnyvale, CA, USA).

### Analyses of cell apoptosis

Effects of GANT61 on apoptosis of T-cell lymphoma cells were evaluated by annexinV- phycoerythrin (PE) /7-aminoactinomycin D (7-AAD) assay. Flow cytometric analysis of these cells labeled with Annexin V- PE) and 7-AAD was performed according to the manufacturer's instructions (BD Biosciences). CLL Cells with designed treatments were harvested, washed twice with cold PBS and resuspended in 1 × binding buffer at a concentration of 1×10^6^ cells/mL. This was followed by transferring 100μL of the solution to a 5 mL tube, to which 5μL of annexin V-PE and 5μL of 7-AAD were added. The tube was gently vortexed and incubated for 15 minutes at room temperature in the dark. At the end of incubation, 400μL of 1 × binding buffer was added. The rates of cellular apoptosis were acquired immediately on a Navios flow cytometer (BECKMAN COULTER). Viable cells are not stained with annexin V-PE or 7-AAD. The necrotic cells were annexin V-PE and 7-AAD-positive, whereas apoptotic cells were annexin V-PE-positive and 7-AAD-negative. After infected with GLI1-RNAi lentivirus or negative control lentivirus, apoptosis of these T-cell lymphoma cells was also detected by this means, untransfected cells served as the control.

### Statistical analysis

Results were expressed as mean ± standard error of mean (SEM). One-way analysis of variance (ANOVA) or t-tests were used to test for differences between groups. Fisher's exact probability test was used to analyze the relationship between the expression of GLI1, p-STAT3 and SOCS3. *P* < 0.05 was accepted as evidence of significance.

## SUPPLEMENTARY MATERIALS FIGURES


