# Supplementary material for: GLI1 inhibitor GANT61 exhibits antitumor efficacy in T-cell lymphoma cells through down-regulation of p-STAT3 and SOCS3
Source: Oncotarget. 2016 Jun 2;8(30):48701–10. doi: 10.18632/oncotarget.9792 (PMC5564718; doi:10.18632/oncotarget.9792)
Supplement: Supplementary file 1 [file oncotarget-08-48701-s001.pdf]

GLI1 inhibitor GANT61 exhibits antitumor efficacy in T-cell lymphoma cells through down-regulation of p-STAT3 and SOCS3

SUPPLEMENTARY FIGURES

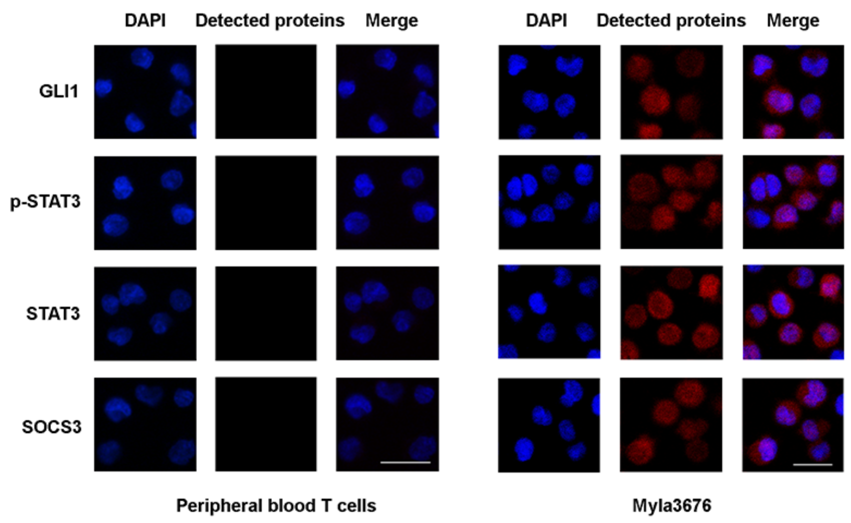

Supplementary Figure S1: The expression of GLI1, p-STAT3, STAT3, and SOCS3 in T-cell line (Myla3676) and peripheral blood T lymphocytes. Immunofluoresce showed elevated expression levels of these indicated proteins in Myla3676 compared to peripheral blood T lymphocytes. The Scale bar=20μm.

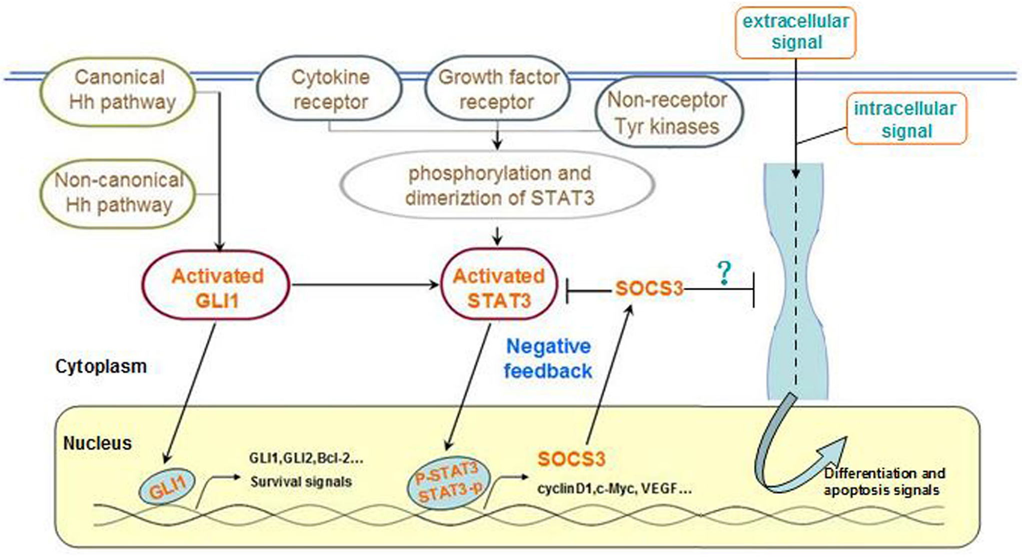

Supplementary Figure S2: The potential relationship of GLI1, STAT3, and SOCS3 in this study. GLI1 serve as a central molecular hub in both canonical and non-canonical Hedgehog pathways. Activated GLI1 subsequently translocate into the nucleus to activate multiple pro-survival target genes ultimately. STAT3 is a well-known mediator activated by various growth factors and cytokines. In the cascade reaction, the phosphorylated STAT3 dimerize and translocate into the nucleus to regulate the transcription of target genes. In addition to genes involved in survival and proliferation, STAT3 induce the transcription of SOCS3 in a negative-feedback manner, leading to the up-regulation of SOCS3. Although the exact regulatory mechanism was quite unknown in T-cell lymphoma, we speculate that the pro-survival or protective potential of Hh/GLI1 signaling is partly mediated by activating STAT3 in T cell lymphomas. Moreover, SOCS3 may participate in tumorgenesis by blocking normal differentiation and apoptosis signals.
